# Supplementary material for: The Molecular Genetics of Dissociative Symptomatology: A Transdiagnostic Literature Review
Source: Genes (Basel). 2022 May 8;13(5):843. doi: 10.3390/genes13050843 (PMC9141026; doi:10.3390/genes13050843)
Supplement: Supplementary file 1 [file genes-13-00843-s001.zip › Table S1.pdf]

Serial number	Search string	PubMed	Scopus	ProQuest
1	Dissociative disorder	420	6046	1172
2	Dissociative disorders	4277	6046	2465
3	Dissociative identity disorder	1078	827	1083
4	Dissociative amnesia	174	453	478
5	Derealization	466	1261	1519
6	Depersonalization	3821	7853	6176
7	Pathological dissociation	116	167	295
8	Genetic	1859318	2878289	1432061
9	Gene	2533638	4066344	1765068
10	(8 OR 9) AND Polymorphism	306083	474500	274827
11	Genome-wide	106408	120326	243598
12	Epigenetic	91480	105873	184785
13	DNA methylation	73948	100435	88283
14	1 OR 2 OR 3 OR 4 OR 5 OR 6 OR 7	8711	14701	9649
15	8 OR 9 OR 10 OR 11 OR 12 OR 13	3508019	5304303	2219171
16	14 AND 15	85	230	273

Table S1: Search strategy for the current review
