# Supplementary material for: The Molecular Genetics of Dissociative Symptomatology: A Transdiagnostic Literature Review
Source: Genes (Basel). 2022 May 8;13(5):843. doi: 10.3390/genes13050843 (PMC9141026; doi:10.3390/genes13050843)
Supplement: Supplementary file 1 [file genes-13-00843-s001.zip › Table S2.pdf]

Reason for exclusion	PubMed	Scopus	ProQuest	Total
**Unrelated**	**46**	**112**	**97**	**255**
Review, dissociation	9	11	10	30
Review, general	3	25	15	43
Original research on dissociation, no genetic component	8	6	18	32
Commentary / editorial / book review	2	3	12	17
Case report	3	1	4	8

Table S2: Numbers of papers excluded and reasons for exclusion from the review
