# Supplementary material for: The Molecular Genetics of Dissociative Symptomatology: A Transdiagnostic Literature Review
Source: Genes (Basel). 2022 May 8;13(5):843. doi: 10.3390/genes13050843 (PMC9141026; doi:10.3390/genes13050843)
Supplement: Supplementary file 1 [file genes-13-00843-s001.zip › Table S4.pdf]

Table S4: Details of standardized measures of dissociative symptomatology used in the studies included in the current review.

Study	Standardized measure of dissociation used?	If yes, details of measure used	Relevant findings, if provided in paper
Lochner et al., 2004	Yes	DES, with cut-off score ≥30 to indicate "high dissociation"	Mean DES 13.6 in OCD, 16.3 in TTM; "high dissociation" in 15.8% OCD, 18.8% TTM; no difference in genetic polymorphism distributions between "high" and "low" dissociators.
Koenen et al., 2005	Yes	PDEQ, PTSD-RI	FKBP5 rs3800373, rs1360870 positively associated with both PDEQ and PTSD-RI scores.
Lochner et al., 2007	Yes	DES, with cut-off score ≥30 to indicate "high dissociation"	Mean DES 13.8 (16.1 in men, 12.5 in women); "high dissociation" in 16.9% OCD; childhood trauma and 5-HTTLPR s/s both positively associated with DES total score.
Savitz et al., 2008	Yes	DES-II, transformed to normal distribution	Mean DES 4.97 for the entire sample (5.70 in BD, 4.71 in relatives); BDNF Met and interaction between COMT Val and childhood trauma positively associated with DES total score.
Tadic et al., 2009	Yes	DES, validated German adaptation; SDQ-20	Mean DES 16.4 in BPD, 2.5 in controls; mean SDQ-20 28 in BPD, 21 in controls; Trend association between SCN9A rs7607967 (G) and SDQ-20 score; possible interaction between rs4371369 (G) and rs4387806 (C), sex, and DES score
McLean et al., 2011	Yes	MCEPS, with cut-off score >3 indicating "significant dissociation"	Association between "pain-vulnerable" COMT haplotype (A_C_C_G) and mean MCEPS score (2.7 vs 2.2 in those with and without haplotype) following trauma.

Pieper et al., 2011	Yes	DES, with cut-off score ≥30 to indicate "high dissociation"	5-HTTLPR s/s genotype associated with higher mean DES scores; 5-HTTLPR s/s more common in those with "high dissociation"
Dackis et al., 2012	Yes	DES-II, transformed to normal distribution	No direct association between FKBP5 CATT haplotype and DES-II score; interaction between FKBP5 CATT haplotype and childhood trauma associated with DES-II score
Schmahl et al., 2012	Yes	DSS	IL6 gene expression positively correlated with DSS total score; IL1B, MAPK1, MAPK3, MAPK8, GNAI2, ARRB1, ARRB2, CREB1 expression negatively correlated with DSS total score.
Wolf et al., 2014	Yes	CAPS items assessing depersonalization and derealization, with frequency score ≥ 1 and intensity score ≥ 2 used to identify "significant dissociation"	No dissociation in 78% of sample; "significant dissociation" in 19%; no genome-wide significant associations; ten suggestive associations with "significant dissociation"; highest peaks at ADCY8 rs263232 and DPP6 rs71534169
Reiner et al., 2016	Yes	CDS	Mean CDS score 45.31 in those with OXTR rs53576 G/G genotype and 28.98 in those with A/A or A/G genotype; trend-level significance.
McCoy et al., 2017	No	-	-
Yaylaci et al., 2017	Yes	A-DES (Adolescent DES), with cut-off score ≥ 4 indicating "pathological dissociation"	Possible negative association between FKBP5 CATT haplotype and A-DES total score, only in those with an early onset and longer duration of maltreatment.
Yu et al., 2017	Yes	HAM-D, item 19 measuring depersonalization / derealization symptoms	Specific group of 19 SNPs associated with lower scores on item 19 of HAM-D; mean score 0.15 in those with the

			specified SNPs, 0.47 in those without.
Honma et al., 2018	Yes	DES, with sub-scores computed for depersonalization / derealization and amnesia symptoms	COMT rs4680 Val/Val genotype associated with total DES score and depersonalization / derealization sub-score but not amnesia sub-score.
Kember et al., 2021	No	-	-
Park et al., 2021	No	-	-

Abbreviations: BD, bipolar disorder; BPD, borderline personality disorder; CAPS, Clinician-Administered PTSD scale; CDS, Cambridge Depersonalization Scale; DES, Dissociative Experiences Scale; DSS, *Dissoziations-Spannungs-Skala*; HAM-D, Hamilton Rating Scale for Depression; MCEPS, Michigan Critical Events Perception Scale; OCD, obsessive-compulsive disorder; PDEQ, Peritraumatic Dissociative Experiences Questionnaire; PPTSD-RI, PTSD Reaction Index; PTSD, post-traumatic stress disorder; SDQ-20, Somatoform Dissociation Questionnaire, 20-item version. Abbreviations used to describe genetic variants are expanded in **Table 1** in the main article.
